# Immune Checkpoint Inhibitors in Uro-Oncology: Urgent Call for Biomarkers

**DOI:** 10.3390/cancers12102768

**Published:** 2020-09-27

**Authors:** Renate Pichler

**Affiliations:** Department of Urology, Medical University Innsbruck, A-6020 Innsbruck, Austria; Renate.Pichler@i-med.ac.at; Tel.: +43-(0)-512-504-24811; Fax: +43-(0)-512-504-28365

Bladder cancer and renal cell carcinoma (RCC) are the second and third most commonly diagnosed cancers in the field of uro-oncology [1]. Immunotherapy has dramatically changed and expanded the therapeutic landscape in both cancer entities [2].

In metastatic RCC (mRCC), immunotherapy is approved both as monotherapy in the second-line setting, according to the CheckMate025 study [3] and in combination (dual checkpoint blockade or checkpoint inhibitor plus vascular endothelial growth factor (VEGF) inhibitor) as first-line (1L) regimes [4,5]. Current guidelines recommend nivolumab plus ipilimumab in intermediate/poor IMDC risk group patients [4], whereas pembrolizumab plus axitinib is indicated in treatment-naïve patients with any IMDC-risk mRCC [5]. In addition, results from other conducted randomized phase III trials have established further combinations (avelumab plus axitinib [6], nivolumab plus cabozantinib (NCT03141177)) in 1L mRCC. Although the superiority of combinations to sunitinib was continuously confirmed in all trials with a benefit in specific small subgroups (intermediate and poor IMDC risk group for nivolumab + ipilimumab [4], and cabozantinib [7]; sarcomatoid RCC for nivolumab + ipilimumab [8], and pembrolizumab + axitinib [9]), the following question remains unanswered for the largest proportion of patients: which combination is the best for which patient? The plethora of novel combined therapeutic regimens in the 1L setting of mRCC is certainly a positive development in the treatment of our patients but poses new challenges in clinical practice. First of all, no head-to-head comparisons of phase III trials are available. Moreover, clinically applicable biomarkers for selecting the right combination for the right patient are still lacking. Thus, an unmet need exists to identify biomarkers in patients with mRCC most likely to respond to specific combined treatments. It has been shown that efficacy of immune checkpoint inhibitors alone or combined with VEGF inhibitors was linked with expression of signatures indicative of myeloid inflammation and T-effector function [10,11,12]. Exploratory biomarker analyses from three clinical trials (CheckMate 214 [13], IMmotion 150 [10], and JAVELIN Renal 101 [6]) were performed but resulted in heterogeneous findings. Concerning the IMmotion 150 trial, angiogenesis, T-effector/IFN-γ response, and myeloid inflammatory gene expression signatures were strongly associated with survival outcomes across treatments (VEGF inhibitors versus checkpoint inhibitors) [10]. Conversely, immune-related gene signatures did not predict progression-free survival (PFS) or overall survival (OS) with nivolumab + ipilimumab [13]. Similarly, as observed in CheckMate 214 [13], molecular biomarker analyses from the JAVELIN Renal 101 trial confirmed that neither expression of PD-L1 nor tumor mutational burden (TMB) had an influence on PFS in both study arms. Interestingly, the development of the “Renal 101 Immunosignature,” including a 26-gene subset, demonstrated longer PFS in the combination arm (avelumab + axitinib) but with no survival influence on sunitinib monotherapy [6]. Validation of this signature was performed using two independent datasets [14,15]. In summary, findings of these exploratory biomarker analyses present the first step towards personalized therapeutic strategies, confirming the immunomodulatory function of antiangiogenic treatment and defining molecular profiles for predicting outcomes with anti-VEGF and immunotherapy. Nevertheless, the step into everyday clinical practice has not yet been taken. 

In metastatic urothelial carcinoma (mUC), various checkpoint inhibitors have revolutionized the therapeutic landscape after a long drought since the introduction of cisplatin-based chemotherapy in the late 1980s [16]. Currently, a novel therapeutic strategy in the 1L setting of mUC, maintenance with avelumab in patients who have not progressed after platinum-based chemotherapy, was introduced by the JAVELIN Bladder 100 trial with astonishing OS data compared to best supportive care alone irrespective of PD-L1 status, cisplatin- or carboplatin-containing chemotherapy and response to 1L chemotherapy [17]. It must be noted critically that a strongly pre-selected group of patients was enrolled in this study (randomization after chemotherapy, the inclusion of only chemotherapy responders, and 4–6 completed cycles of chemotherapy) [17]. Another therapeutic concept is to combine primary chemotherapy immediately with immunotherapy in all (chemotherapy-naive) patients with mUC. Whereas first interim analyses confirmed that the KEYNOTE-361 and DANUBE trial did not meet their primary endpoints of improving PFS and OS versus chemotherapy alone [18,19], IMvigor130 is the first immune checkpoint inhibitor study demonstrating improvement in PFS over chemotherapy alone. Nevertheless, interim OS data did not cross the pre-specified interim efficacy boundary for statistical significance [20]. Exploratory biomarker analyses from IMvigor130 provide the first evidence that specific subgroups (IC2/3, TMB^high^, APOBEC mutagenesis^high^, IC2/3 + TMB^high^) possibly benefit from atezolizumab monotherapy [21]. So it will remain exciting to observe whether TMB [22], APOBEC mRNA expression profiling [23], next-generation sequencing of cell-free circulating tumor DNA [24], and the Cancer Genome Atlas (TGCA) molecular subtyping [25] will definitely bring light into the darkness of biomarker research in mUC.
